# Quality assurance of an established online adaptive radiotherapy program: patch and software upgrade

**DOI:** 10.3389/fonc.2024.1358487

**Published:** 2024-05-28

**Authors:** Nema Bassiri, John Bayouth, Michael D. Chuong, Rupesh Kotecha, Yonatan Weiss, Minesh P. Mehta, Alonso N. Gutierrez, Kathryn E. Mittauer

**Affiliations:** ^1^ Department of Radiation Oncology, Miami Cancer Institute, Baptist Health South Florida, Miami, FL, United States; ^2^ Herbert Wertheim College of Medicine, Florida International University, Miami, FL, United States; ^3^ Department of Radiation Medicine, Oregon Health and Science University, Portland, OR, United States

**Keywords:** quality assurance, software upgrade, adaptive radiotherapy, treatment planning system, end-to-end testing, MR linear accelerator

## Abstract

**Introduction:**

The ability to dynamically adjust target contours, derived Boolean structures, and ultimately, the optimized fluence is the end goal of online adaptive radiotherapy (ART). The purpose of this work is to describe the necessary tests to perform after a software patch installation and/or upgrade for an established online ART program.

**Methods:**

A patch upgrade on a low-field MR Linac system was evaluated for post-software upgrade quality assurance (QA) with current infrastructure of ART workflow on (1) the treatment planning system (TPS) during the initial planning stage and (2) the treatment delivery system (TDS), which is a TPS integrated into the delivery console for online ART planning. Online ART QA procedures recommended for post-software upgrade include: (1) user interface (UI) configuration; (2) TPS beam model consistency; (3) segmentation consistency; (4) dose calculation consistency; (5) optimizer robustness consistency; (6) CT density table consistency; and (7) end-to-end absolute ART dose and predicted dose measured including interruption testing. Differences of calculated doses were evaluated through DVH and/or 3D gamma comparisons. The measured dose was assessed using an MR-compatible A26 ionization chamber in a motion phantom. Segmentation differences were assessed through absolute volume and visual inspection.

**Results:**

(1) No UI configuration discrepancies were observed. (2) Dose differences on TPS pre-/post-software upgrade were within 1% for DVH metrics. (3) Differences in segmentation when observed were small in general, with the largest change noted for small-volume regions of interest (ROIs) due to partial volume impact. (4) Agreement between TPS and TDS calculated doses was 99.9% using a 2%/2-mm gamma criteria. (5) Comparison between TPS and online ART plans for a given patient plan showed agreement within 2% for targets and 0.6 cc for organs at risk. (6) Relative electron densities demonstrated comparable agreement between TPS and TDS. (7) ART absolute and predicted measured end-to-end doses were within 1% of calculated TDS.

**Discussion:**

An online ART QA program for post-software upgrade has been developed and implemented on an MR Linac system. Testing mechanics and their respective baselines may vary across institutions, but all necessary components for a post-software upgrade QA have been outlined and detailed. These outlined tests were demonstrated feasible for a low-field MR Linac system; however, the scope of this work may be applied and adapted more broadly to other online ART platforms.

## 1 Introduction

Online adaptive radiotherapy (ART) is an emerging paradigm that is becoming routine for MR Linacs and X-ray-based image-guided radiotherapy (RT) systems. The assessment of the accuracy and workflow validation is critical to the implementation and ongoing performance of ART platforms. Currently, there are many studies describing the commissioning of online adaptive radiotherapy through end-to-end testing and deformable image registration evaluation (1–4). However, there is limited literature to date on routine quality assurance (QA) for online adaptive RT, specifically regarding patch or software upgrades for established programs. Software upgrades affect the front-end of the patient workflow, specifically the treatment planning system (TPS) and its ability to function in the context of ART. Additionally, a software upgrade would also impact the delivery console, which includes a built-in TPS for online ART. Both the TPS and the delivery console are integrated in the ART workflow, and their performance needs to be tested (5). The need for QA recommendations pertaining to updating online ART platforms is important for the maintenance feasibility in addition to ongoing quality and safety.

The success of an online ART program relies on the ability of the delivery console to receive a baseline plan from the TPS, adapt the plan to the anatomy of the day, and create a new adaptive plan. As such, integral workflow steps include (1) propagate (deformably and/or rigidly) organs at risk (OARs) and target volumes to the anatomy of day (6); (2) recreate planning-dependent structures based on newly modified OARs and target volumes (7); (3) generate the ideal fluence and plan quality per the anatomy of the day; and (4) send over updated delivery instructions to the Linac. The purpose of this work is to describe the necessary tests to perform after a standard software patch installation and/or upgrade for an established online ART program. Specifically, the proposed set of tests are designed to evaluate the delivery console and TPS systems’ integration with one another to ensure high-quality online ART after a system patch and/or software upgrade.

## 2 Materials and methods

### 2.1 Scope

It is assumed that the online ART system being upgraded is fully commissioned and has been treating patients prior to the upgrade and/or patch. The focus of the outlined tests was performed on the MRIdian system (ViewRay Systems Inc., Oakwood, OH). Note that figures and tables included in this work are specific to the MRIdian platform; user interface (UI)-specific details have been included for demonstration purposes. A patch installation refers to an update that resolves a software bug, and a software upgrade refers to a functionality update in the UI. Note that for this work, post-software upgrade refers collectively to both a patch installation and software upgrade.

### 2.2 Overview

#### 2.2.1 ART platform overview

The MRIdian system (A3i, version: 5.5.4.14) has its own proprietary planning system which is bifurcated into two distinct platforms (1): the MRIdian TPS which is used during the initial planning stage and (2) the MRIdian treatment delivery system (TDS) which is a TPS integrated into the delivery console for online ART planning. Specifically, the initial plan is created on the TPS and is loaded to the TDS for use as the baseline plan for online ART. Details of the MRIdian ART workflow have been previously described (2, 6, 8).

#### 2.2.2 Overview of tests

An overview of the tests performed for online adaptive post-software upgrade at our institution is outlined in **Table 1**. A brief overview of the QA procedure, materials utilized (i.e., UI or physical phantom), estimate of full time equivalent (FTE) hours, and type of results (i.e., functional or dosimetric) are presented for each test.

**Table 1:** Overview of the online adaptive radiotherapy post-software upgrade QA tests, materials required, anticipated hours, and description of results.

ART post- software upgrade QA procedure	Overview	Materials	FTE time (h)	Description of results
Configuration consistency	Checksum on UI configuration	TDS	0.5	System settings verification
TPS beam model consistency	Compare dosimetry of a clinical plan pre- and post-software upgrade	TPS	1.0	Dosimetric comparison for relevant DVH metrics
Sub-end-to-end: Segmentation consistency	Planning structures were created on a phantom and compared using TPS and TDS	MR phantom TPS, TDS	2.0	Comparison of contour volumes and visual agreement
Sub-end-to-end: Dose calculation consistency TPS vs. TDS	Compare dose of an SBRT plan calculated on the TPS vs. TDS	MR phantom TPS, TDS	1.0	Comparison of dose calculated by TPS and TDS for same fluence
Sub-end-to-end: Optimizer robustness consistency TPS vs. TDS	Run ART workflow with a phantom, and compare baseline TPS plan with a newly reoptimized ART plan	MR phantom TPS, TDS	1.5	Comparison of the modulation and dose differences of ART plan versus baseline TPS plan
Sub-end-to-end: CT density table consistency	Run ART workflow and compare electron density on TDS with TPS	MR phantom TPS, TDS	0.5	Comparison of electron density values from TPS and TDS
Full-end-to-end: Absolute dose motion phantom	Full end-to-end test with introduced target shifts	MR motion phantom ionization chamber TDS	5.0 (four end-to-end procedures at 1 h per procedure + simulation and initial plan generation)	Comparison of TDS calculated dose with measured dose from ionization chamber

### 2.3 QA procedures

#### 2.3.1 Configuration consistency

It is important to evaluate the consistency of the UI settings after a software upgrade. For this evaluation, we generated a UI configuration report to evaluate the checksum differences between dates pre- and post-software upgrade. The UI settings generated from this report include all MRIdian system computers including TDS, TPS, MR host, Linac control computer (LCC), treatment delivery computer unit (TDCU), core, and database. Any differences are noted by the configuration value and are highlighted to indicate a difference.

#### 2.3.2 TPS beam model consistency

For evaluating the consistency of the beam model and the dose calculation algorithm, it is recommended by MPPG 5a (section 9) to compare the dosimetry pre- and post-software upgrade on a benchmark plan (9). For this evaluation, we used an existing clinical case and recalculated the plan post-software upgrade. We then compared the relevant dose volume histogram (DVH) metrics (i.e., D0.5 cc, D95% etc.) of the clinical case to the pre-software upgrade respective DVH metrics.

#### 2.3.3 Sub-end-to-end: segmentation consistency (rigid assessment)

The ability to accurately propagate rigid segmentation and contour dependent expansions and Boolean logic from the initial treatment plan onto the anatomy of the day is crucial for ART. Rigid propagation is important for delineation of the targets (gross tumor volume [GTV] and clinical target volume [CTV]) on the anatomy of the day as a starting point per our institutional ART workflow and planning technique (8, 10). As such, we used a rigid phantom with predefined Boolean segmentation logic to evaluate the segmentation consistency between the baseline plan and the adaptive plan.

To this end, we scanned an MR-compatible motion phantom (QUASAR, Modus Medical Devices Inc., Ontario, Canada) and followed our institutional MR simulation workflow. MR simulation was performed using the 3D true fast imaging with steady-state free precision (TrueFISP) imaging protocol. Since voxel size has dependency on how regions of interest (ROIs) are interpolated/expanded, we thought it was important to test all imaging protocols with the institutional segmentation technique. The imaging protocols evaluated included our institutional abdominal and thoracic protocol of 50 × 50 × 35.8 cm^3^ with a resolution of 1.5 mm^2^ in-plane and slice thickness of 3 mm, and our pelvic protocol of 50 × 50 × 35.8 cm^3^ with isotropic 1.5-mm^3^ resolution.

The institutional segmentation technique will be briefly described here for context for the ROI consistency verification test. Key features were segmented including OARs and a GTV. Our institutional planning approach utilizes a set of rules which creates dependent planning structures. The following target-derived planning ROIs were created based on these predefined rules in the TPS: (1) planning target volume (PTV) was created as a 0.3 cm uniform expansion of the GTV; (2) “Ring_2cm” was created as a 1 cm-diameter shell that is a 2 to 3 cm expansion of the PTV; (3) “LowDoseRing” was created by subtracting the 3 cm PTV expansion from the external contour; and (4) “GTV_core” was created to drive the hotspot in the optimizer and was a uniform contraction of the “GTVopt” by 0.3 cm. An identical set of planning structures should automatically be created when the registered phantom is loaded onto the TDS. Our institutional planning approach is based on summing then expanding OARs and subtracting the intersection between them and target structures using “rules”. The OARs of interest were summed into a single structure (“AllOARs”). This large, contiguous structure was then uniformly expanded by 0.3 cm to create planning-at-risk volume (“AllPRVs”) which was used to carve out any overlapping target structures. The remaining targets (“GTVopt” and “PTVopt”) are used to direct the prescription dose when optimizing the plan, by subtracting “AllPRVs” from the respective GTV and PTV.

**Figure 1:**
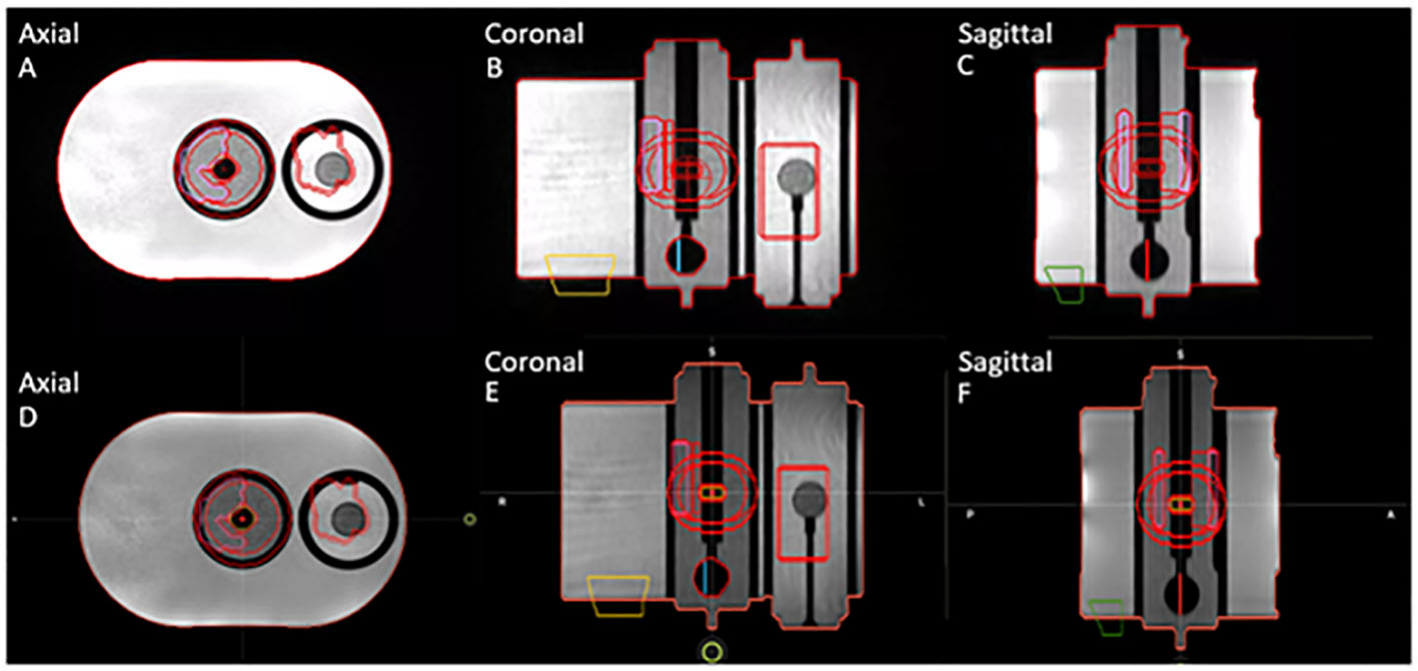
Segmentation consistency between baseline plan on TPS **(A–C)** and adaptive plan on TDS **(D–F)** for axial, coronal, and sagittal planes of QUASAR phantom and contours of targets and OARs.

For this segmentation consistency check, all structures were set to rigid on the baseline plan in the TPS. The same TrueFISP MR scan from simulation was acquired in the adaptive workflow on the TDS, and the ROIs were then compared. Specifically, the geometric dimensions and locations of the contours and planning-dependent outputs were both visually and quantitatively evaluated between the TPS and the TDS. **Figure 1** displays the axial, coronal, and sagittal planes of the MR motion phantom with all targets and OARs on the TPS baseline plan (A-C) and the TDS adaptive plan (D-F).

#### 2.3.4 Sub-end-to-end: dose calculation consistency TPS vs. TDS

Note that since the online ART environment is a different UI than the initial plan creation, as previously described, evaluating the consistency between the dose calculation algorithm is important. For this measurement, we used the same phantom setup from the segmentation consistency test. A stereotactic body radiotherapy (SBRT) treatment plan was designed on the phantom setup using the TPS on the simulation dataset, as previously described.

**Figure 2:**
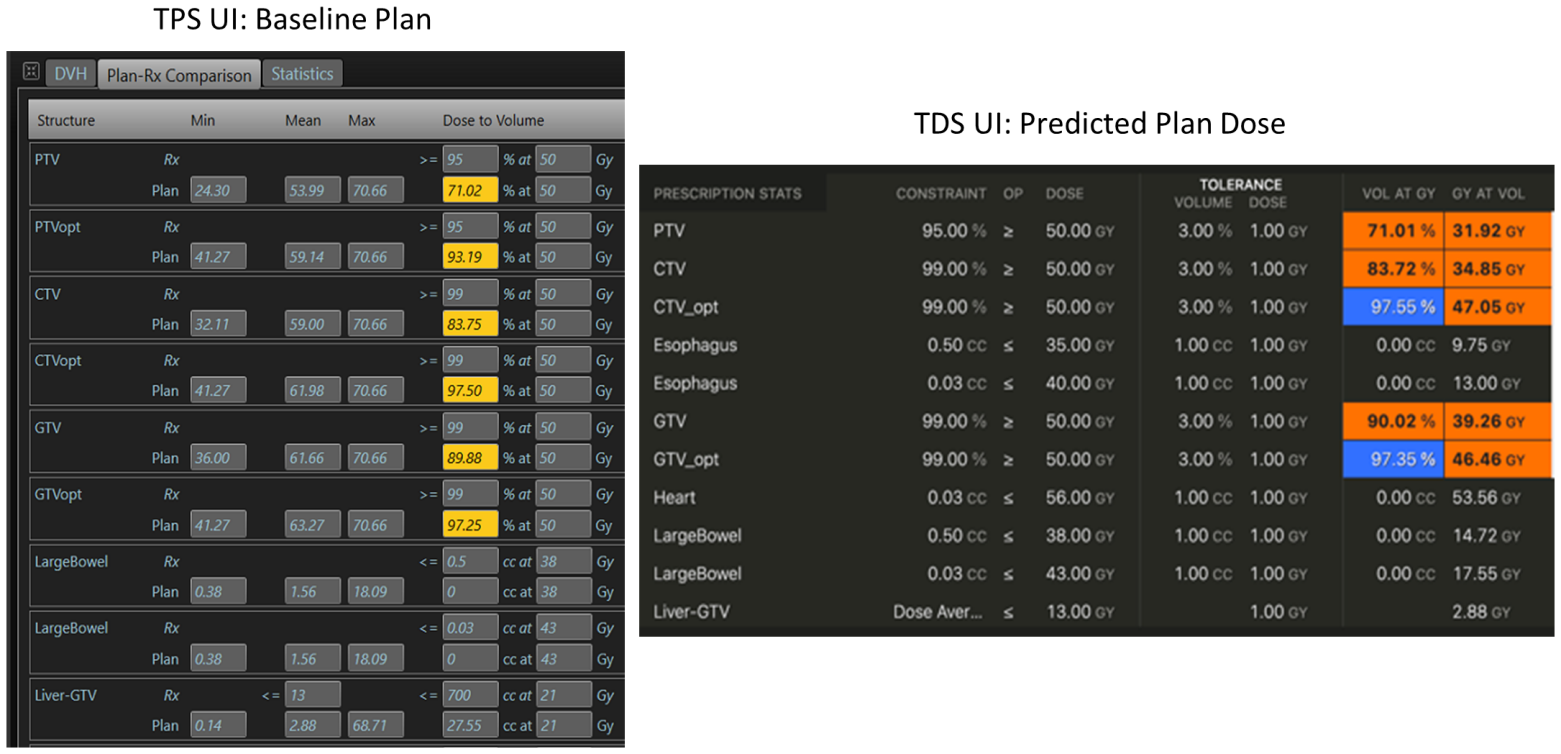
Example of dose calculation consistency check between the TPS (left) during the baseline planning, and the TDS (right) during the ART planning for the same plan/fluence.

We then evaluated the differences in the TPS baseline plan’s dose distribution to the “predicted dose” calculation from the TDS’ adaptive workflow. Since the geometry and ROIs of this evaluation were rigid, the DVH metrics can be simply evaluated to determine consistency of the dose calculation algorithm on the TDS to the TPS. Note that the predicted dose is the fluence of the simulation baseline plan from the MRIdian TPS recalculated onto the image acquired at the time of online ART. In short, the dose distribution from the “predicted plan” should match that of the initial TPS generated plan within Monte Carlo statistical uncertainty. **Figure 2** displays an example of the dose statistics for the baseline plan in the TPS UI (left) and the predicted plan in the TDS UI (right) used to evaluate dose calculation consistency using identical beam fluence.

Furthermore, gamma analysis could be used to compare the consistency between two calculation-generated dose distributions. DICOM dose files of the TPS plan and the predicted plan from the TDS were exported to SNC Patient software (Sun Nuclear, Melbourne, FL). Gamma indices of 2%/2 mm and 1%/1 mm were used to assess the difference between the dose distributions generated from the TPS and TDS of the same fluence.

#### 2.3.5 Sub-end-to-end: optimizer robustness consistency TPS vs. TDS

**Figure 3:**
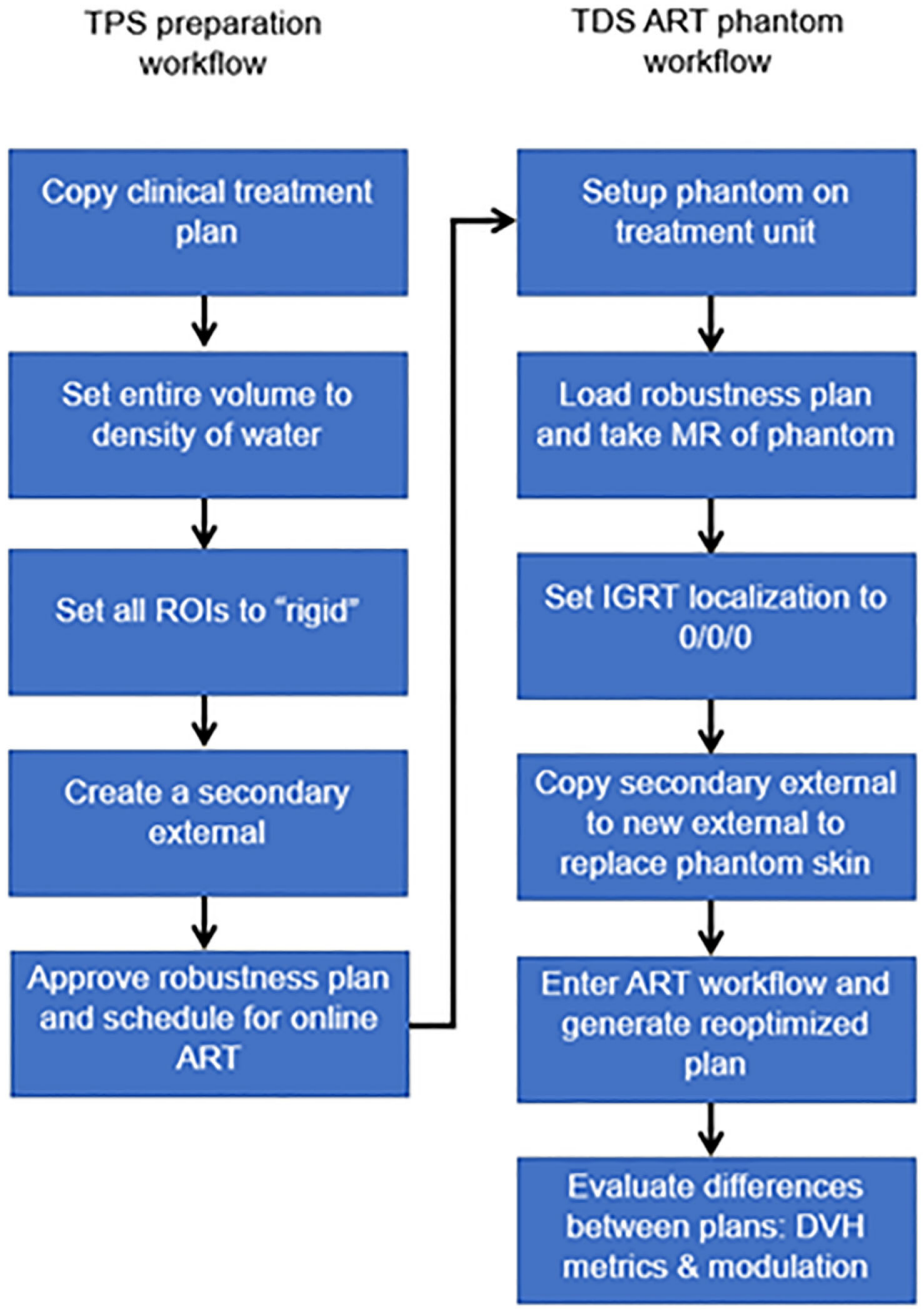
Overview of workflow to evaluate the optimizer robustness consistency between the baseline plan in the TPS and adaptive plan in the TDS.

For online ART, evaluating the differences in the optimizer performance (i.e., reproducibility) between the TPS and TDS is important to benchmark. Any difference if not understood can lead to challenges in the online adaptive replanning quality and efficiency. As such, we evaluated the differences of the optimizer and leaf sequencer between the TPS and the TDS. **Figure 3** displays the workflow/method for evaluating optimizer robustness between the TPS and TDS. Note that a clinical plan can be used following TPS preparation as outlined below; the ART workflow is then performed on an MR-compatible phantom.

Any previously treated clinical case can be selected for this exercise. All ROIs were set to a rigid propagation. The CT that was used to map the electron density for the clinical case was removed, and all densities were overridden to match that of water. Note that the accuracy of the electron density is not important for this test. What we are measuring is the ability to replicate a plan and creating a homogenous treatment volume simplifies this procedure. A separate external contour was created on the initial patient image and set as a rigid structure in the MRIdian TPS.

The multimodality abdominal phantom was then set up to the lasers and imaged using our standard institutional imaging protocol. Note that any MR-imageable phantom can be used for this assessment, since the density is already predefined as rigid ROI and the electron density is homogenous water. The skin generated by the TDS was replaced with the rigid external ROI created during treatment planning. The “fully reoptimized plan” on the TDS was then compared with the original plan (i.e., original fluence plan created by the TPS calculated on the simulation baseline anatomy). Robustness is evaluated based on the similarity between the original from the TPS generated dose distribution and the ART plan generated by the TDS optimizer. The MU and number of segments were compared with evaluate the leaf sequencer similarity.

#### 2.3.6 Sub-end-to-end: CT density table consistency

**Figure 4:**
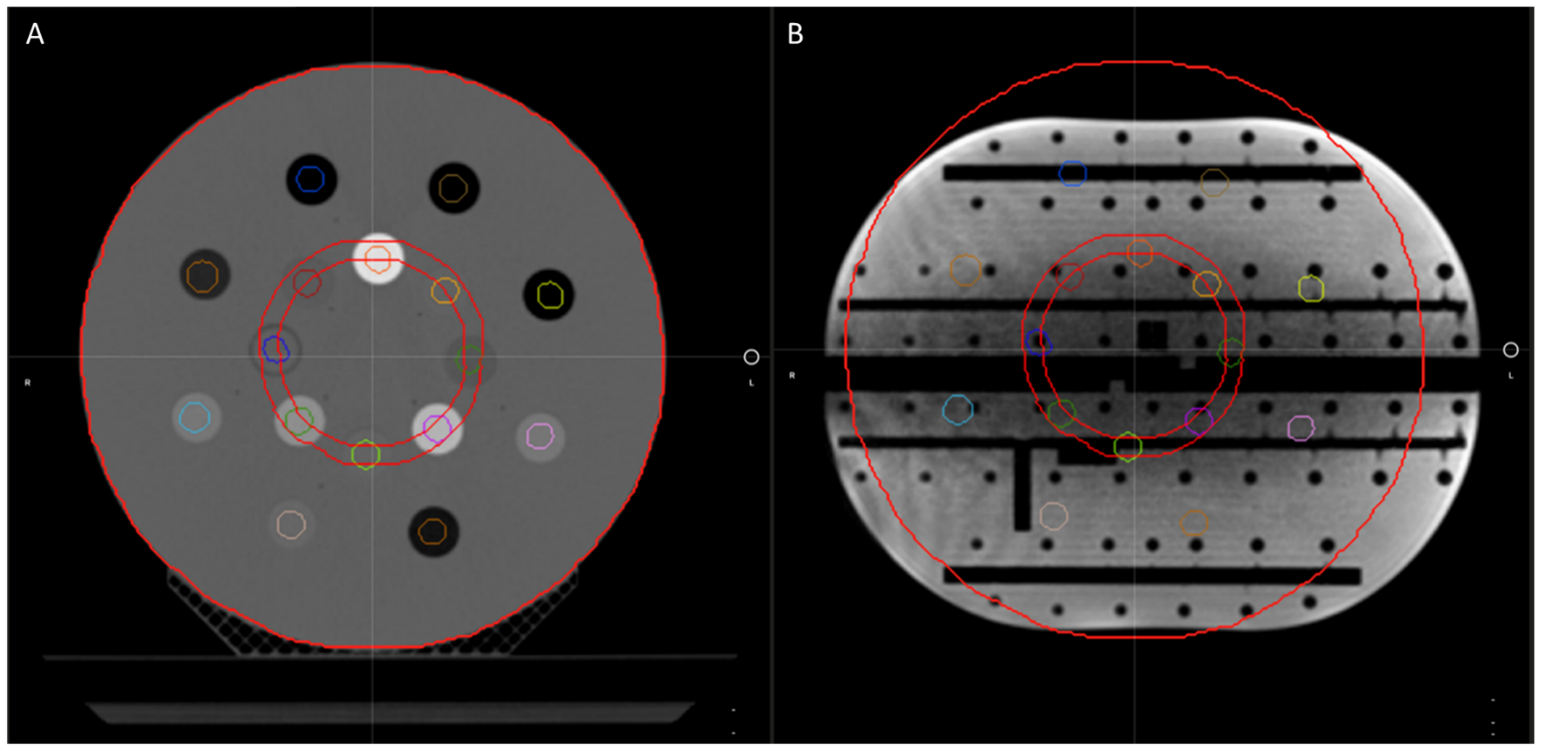
The CT electron density phantom as it appears on the TPS **(A)** and the CT electron density phantom projected onto the Magphan in the ART workflow for verification of image value consistency **(B)**.

To verify that the CT density table in the TDS is consistent with the density table of the TPS, the electron density propagation for relevant values should be verified. For this assessment, we used a heterogeneous phantom with known density plugs (CT Electron Density Phantom, Sun Nuclear, Melbourne, FL) that had been previously CT scanned. Since such CT density phantom is not capable of generating MR signal, we used an MR-imaging phantom (Magphan RT 820, Phantom Laboratory, Greenwich, NY) that approximated a similar size of the density phantom. The MR-imaging phantom was then used as the primary dataset of the initial plan for the TPS validation and for the primary dataset of the ART plan for TDS validation. **Figure 4** displays the CT electron density phantom as it appears on the TPS (A), and the CT electron density phantom projected onto the MR-imaging phantom in the ART workflow (B). The electron density values relative to water as displayed in the UI were compared.

#### 2.3.7 Full-end-to-end: absolute dose motion phantom

The ability to dynamically adjust target contours, derived Boolean structures, and ultimately the optimized fluence is the end goal of ART. We developed an end-to-end test to assess the dosimetric accuracy of an RT plan which had its target volume simulating interfractional motion. Our test was designed to enable statistical differences in the predicted dose compared with the reoptimized adaptive dose, by ensuring the motion of the target was beyond the original fluence (i.e., adapted target was positioned beyond the treatment volume of the original plan). We designed this test in the same manner as our ART workflow (i.e., fully reoptimized with normalization); this will vary based on the institutional ART technique. Plan design should reflect institutional technique and methods will vary.

**Figure 5:**
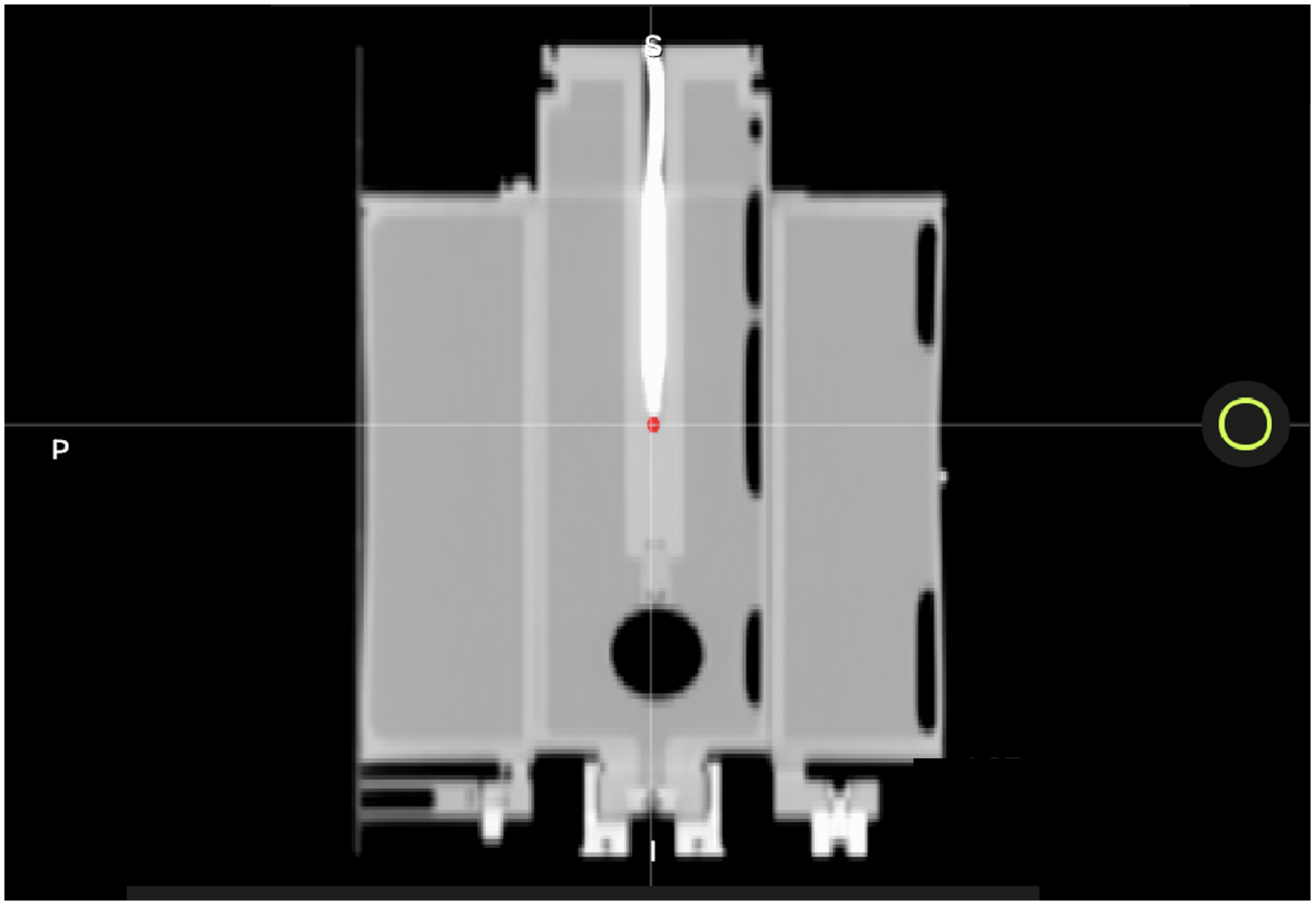
A CT image of the QUASAR with A26MR ionization chamber. The A26MR active volume is contoured in red.

We used an MR-compatible motion phantom (QUASAR) and an Exradin A26MR ionization chamber (0.015 cc, Standard Imaging Inc., Middleton, WI) to perform this test. The movable phantom plunger (i.e., chamber holder) was initially positioned at the positive peak position of its travel path, +19.9 mm. Our standard clinical imaging protocol was used, and a previously acquired CT scan of the phantom was imported into the TPS to map the electron density. **Figure 5** displays the CT of the motion phantom with the A26MR chamber.

We created a treatment plan using the aforementioned planning technique by creating a GTV volume of 2 cm in diameter and centered on the active volume of the ionization chamber. The GTV was uniformly expanded, planning rings were generated, and custom OARs were used to create AllOARs, AllPRVs, GTVopt, and PTVopt structures.

The active volume of the chamber was contoured to compare the calculated dose to the measured dose. The generated plan was designed to deliver a homogenous dose distribution across the PTV to avoid large dose gradients and reduce measurement uncertainty. As such, for the baseline plan, the maximum/minimum/mean dose to the PTV and the contoured A26MR active volume on the TPS were 8.40/7.95/8.20 and 8.29/8.21/8.24 Gy per fraction, respectively.

**Figure 6:**
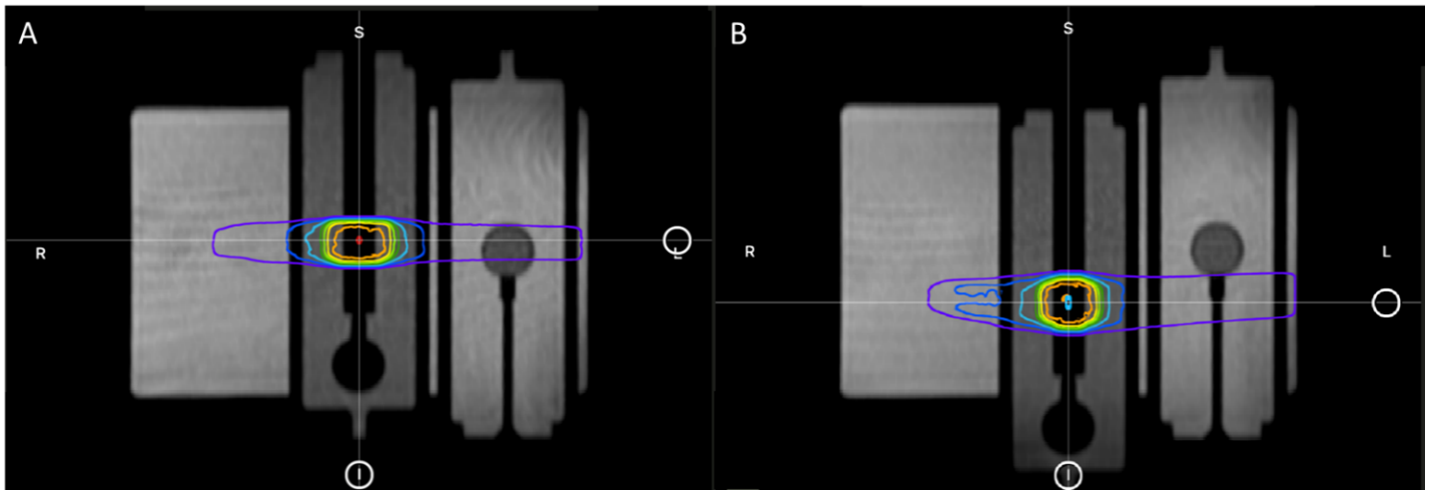
The plunger at its initial +19.9 mm position for baseline plan **(A)** and the shifted −19.9 mm position for adaptive plan **(B)**.

Without moving or touching the phantom, the motion phantom’s plunger was positioned to the other extreme position (−19.9 mm) using the phantom’s software. Per the ART workflow, a new MR scan was acquired for the anatomy of the day and the GTV was adapted to the new location of the A26MR. Specifically, the GTV and A26 active volume contour were shifted longitudinally by the fixed amount the motion phantom was translated (+19.9 mm to −19.9 mm). **Figure 6** displays the motion phantom shifting between the two positions with respective dose distribution from the baseline plan (A) and adaptive plan (B).

All subsequent derived structures were recreated based on the adapted GTV through predefined Boolean logic and expansions. Note that the active volume contour of the A26MR was not a derived structure; it was created manually.

There are two treatment techniques that we tested: replicating the baseline plan onto the original geometry of +19.9 mm position (predicted plan) and adapting the baseline plan onto the new geometry of −19.9 mm position (ART plan). Both treatment techniques were delivered as a continuous treatment and an interrupted treatment, where the beam and MRI were completely turned off before the phantom was reimaged and treatment was set to resume. For each treatment, the measured and calculated dose to the A26MR was compared. For the predicted plan, we also compared the calculated statistics of the TPS and TDS to the delivered dose, as a secondary evaluation of the TPS to TDS dose calculation consistency.

## 3 Results

### 3.1 Configuration consistency

**Figure 7:**
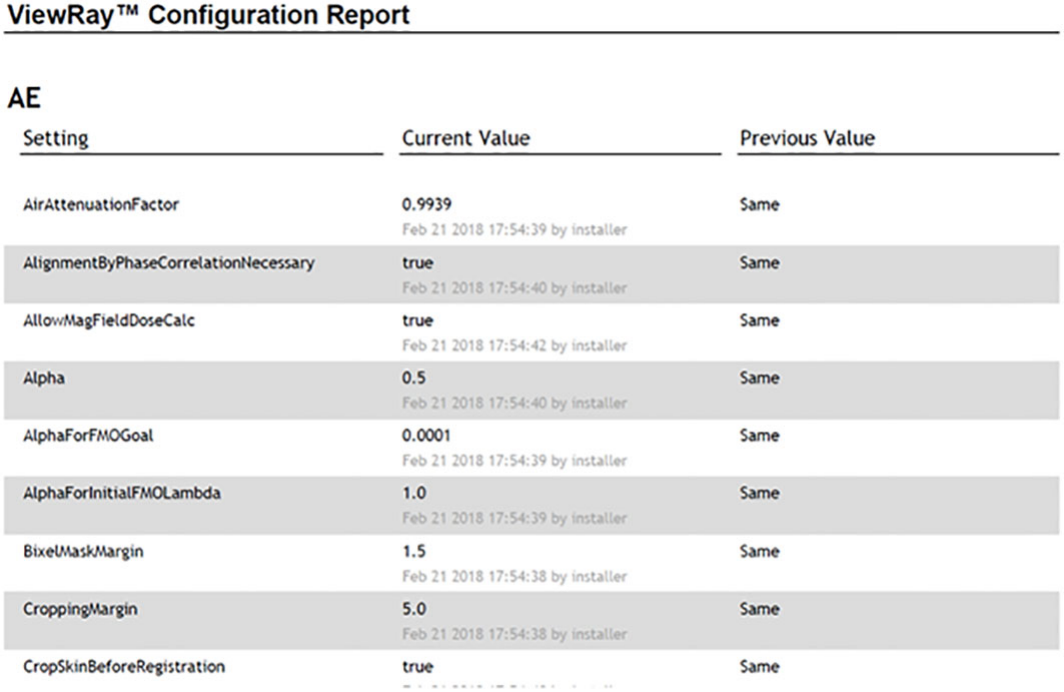
An example of the MRIdian configuration report with post-software upgrade and pre-software upgrade UI settings shown in the “current value” and “previous value,” respectively.

For the configuration consistency, no differences were observed in the UI configuration report. **Figure 7** demonstrates an example of the MRIdian configuration report. Note that the UI configurations were maintained, and UI settings were confirmed pre-software upgrade to post-software upgrade.

### 3.2 TPS beam model consistency

For the TPS beam model consistency, the results between pre- and post-software upgrade dose distributions are shown in **Table 2** for the same case. Note that both percentage of volume and absolute volume statistics were evaluated. The large percentage differences in point dose metrics (i.e., V40 Gy (cc) to small bowel) are inflated as the TPS calculates the 40 Gy dose to the small bowel as 0.03 cc (pre-software upgrade) versus 0.04 cc (post-software upgrade), a 33.33% increase which is equivalent to a 0.01 cc increase. The slight differences in point doses receiving a given volume are not clinically significant and within the Monte Carlo dose calculation uncertainty.

**Table 2:** TPS beam model consistency results pre- and post- software upgrade with relevant dose volume statistics.

ROI	Metric	Pre-software upgrade	Post-software upgrade	Difference
PTV	V40 Gy (%)	91.10	91.33	0.25%
PTVopt	V40 Gy (%)	98.41	98.72	0.32%
GTV	V50 Gy (%)	97.85	97.77	−0.08%
GTVopt	V50 Gy (%)	99.98	100	0.02%
Duodenum	V40 Gy (cc)	0.07	0.08	0.01 cc
Duodenum	V35 Gy (cc)	0.50	0.53	0.03 cc
Small bowel	V40 Gy (cc)	0.03	0.04	0.01 cc
Small bowel	V35 Gy (cc)	0.14	0.15	0.01 cc

### 3.3 Sub-end-to-end: segmentation consistency (rigid assessment)

For the segmentation consistency, the differences between the contour volumes of the TPS and TDS are shown in **Table 3** for the same case. The results were consistent, and there were no clinically impactful differences. Of note, there were slight volume differences when structures were expanded.

**Table 3:** Segmentation consistency results of TPS versus TDS with relevant volumes statistics.

ROI	TPS volume (cc)	TDS volume (cc)	Absolute difference (cc)
PTV	6.39	6.39	0.00
PTVopt	5.43	5.43	0.00
GTV	2.86	2.86	0.00
GTVopt	2.58	2.58	0.00
Ring2cm	165.78	166.80	1.02
OAR1	104.27	104.27	0.00
OAR2	162.27	162.27	0.00
PTV+2cm	128.89	126.98	−1.91
PTV+3cm	294.67	293.78	−0.89

### 3.4 Sub-end-to-end: dose calculation consistency TPS vs. TDS

**Figure 8:**
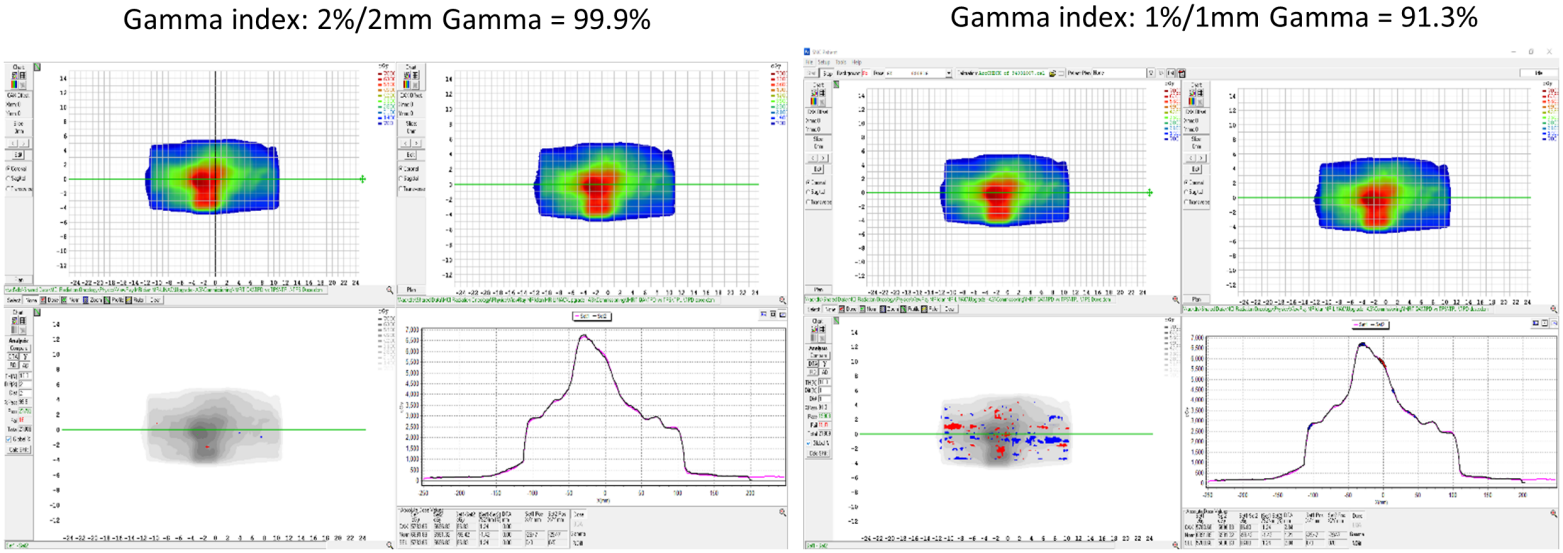
The dose calculation consistency between the TPS and TDS with gamma comparison using an index of 2%/2 mm and 1%/1 mm.

For the dose calculation consistency between the TPS and TDS, a plan created by the TPS was compared with the predicted plan of the TDS. **Figure 8** displays the gamma comparison between the two dose distributions using an index of 2%/2 mm and 1%/1 mm. The passing rates were 99.9% and 91.3% for the 2%/2 mm and 1%/1 mm indices, respectively.

### 3.5 Sub-end-to-end: optimizer robustness consistency TPS vs. TDS

For the robustness check between the TPS and TDS, a patient case was applied through an online ART workflow to compare the TPS generated plan to the fully reoptimized online ART-generated plan. **Table 4** contains the results of the robustness consistency with relevant clinical dose volume goals and modulation parameters displayed for both plans. Maximum percentage and absolute volume differences in dose were 2.01% and 0.56 cc for respective CTV V33 Gy and small bowel V35 Gy, demonstrating that online adaptive plan quality was upheld using the institutional planning technique on a rigid geometry.

**Table 4:** Optimizer robustness consistency as shown through plan quality metrics of a clinical plan created by the TPS and compared with the fully reoptimized online ART plan on the TDS.

ROI/modulation	Metric	TPS initial plan	Online ART plan	Difference
PTV50	V50 Gy (%)	95	95	0.00%
PTV50opt	V50 Gy (%)	95.31	95.27	−0.04%
GTV	V50 Gy (%)	100	100	0.00%
PTV33	V33 Gy (%)	95.51	93.97	−1.61%
PTV33opt	V33 Gy (%)	96.53	95.83	−0.73%
CTV	V33 Gy (%)	99.52	97.52	−2.01%
Small bowel	V35 Gy (cc)	0.19	0.75	0.56
Stomach	V35 Gy (cc)	0	0	0
Large bowel	V38 Gy (cc)	0.03	0	−0.03
Modulation	# of beams	16	16	0
Modulation	# of segments	49	50	+1
Modulation	# MUs	2,883.5	3,925.4	136%
Modulation	MU/prescribed dose	2.88	3.93	136%

### 3.6 Sub-end-to-end: CT density table consistency

The electron density value comparison of the deformed density phantom was performed in the TPS and TDS for the validation of the image value to density conversion in the initial plan workflow and online adaptive workflow, respectively. **Table 5** displays the measured relative electron density values from the TPS and TDS comparison. Note that the maximum difference observed was the lung at 3.57%; however, 0.28 and 0.29 relative electron densities between TPS and TDS are negligible.

**Table 5:** Comparison of the relative electron density of known value, TPS, and TDS.

ROI	Known electron density relative to water	Relative electron density (TPS)	Relative electron density (TDS/Online ART)	% difference TPS to known density	% difference TPS to TDS
Air	0	0.05	0.05	–	0.00%
Lung	0.29	0.28	0.29	−3.45%	3.57%
Water	1	0.99	0.97	−1.00%	−2.02%
Bone	1.28	1.26	1.24	−1.56%	−1.59%
Dense Bone	1.69	1.67	1.66	−1.18%	−0.60%

### 3.7 Full-end-to-end: absolute dose motion phantom

Simulated interfractional motion on an MR-compatible phantom was performed as an end-to-end absolute dose procedure. Four end-to-end permutations as shown in **Table 6** were performed: (1) predicted dose, (2) predicted dose with interruption, (3) ART dose, and (4) ART dose with interruption. A predicted dose plan and the ART fully reoptimized plan were generated based on the nominal anatomy and induced anatomical change, respectively. Note that the predicted dose plan was performed in the adaptive workflow to confirm that the original fluence delivery parameters would maintain in an ART environment when the non-ART plan was selected for delivery. All dosimetric results (**Table 6**) were within 1% agreement of measured to calculated values. Note that for the interruption tests, approximately 60% of MU were delivered prior to the planned interruption.

**Table 6:** The end-to-end results of calculated and measured dose for online adaptive workflow on initial and shifted anatomy.

End-to-end evaluation	Phantom geometry	TDS calculated dose (Gy)	Measured dose (Gy)	% difference (calculated to measured dose)
Predicted dose	Unshifted	8.22	8.22	0.00%
Predicted dose with interruption	Unshifted	8.22	8.20	−0.17%
ART dose	Shifted	13.82	13.74	−0.58%
ART dose with interruption	Shifted	13.95	13.82	−0.90%

## 4 Discussion

The purpose and key advantage of ART is the ability to adjust contours and their derived planning-dependent structures to the daily anatomy, such that a newly optimized fluence can maximize the therapeutic ratio. The aim of this work is to describe the necessary tests to perform after a software upgrade has been implemented to an ART-capable system. It is assumed that the system being upgraded is already commissioned and used in a clinical environment. Our tests were performed on a MRIdian system; however, the processes outlined in this work can be adapted and applied to other systems capable of online ART. Note that QA tests should be designed to validate elements of the UI that were impacted by the software upgrade in conjunction to online ART process and workflow procedures. As such, there may be QA elements outside the scope of this work that may be relevant in the QA of an online ART post-software upgrade (i.e., image quality).

The UI configuration report was generated in real-time after patch installation and was compared with institutional-specific approved configurations. It is expected that different systems and institutions will have different baseline parameters. The purpose of the UI configuration report is to ensure and verify to the user that all system settings are maintained. It is possible that not all system configurations are included in the vendor-supplied report, and users should work with their respective vendor to confirm if additional configuration files need to be manually verified for integrity.

When analyzing the segmentation consistency, we observed slight differences in the volumes for the output of the expansion-based rule dependent ROIs. These differences were observed at the interface of a voxel and likely due to the non-partial voxelization of the MRIdian system. The translation of rigid ROIs maintained the integrity between TPS and TDS during the online ART workflow.

When comparing the TPS’s beam model before and after the software upgrade, dosimetric agreement (**Table 2**) was found to be consistent to the statistical uncertainty of 0.5% of the Monte Carlo histories. Note that the DVH metrics for large volumes such as target coverage metrics were within the accepted uncertainty of Monte Carlo. However, slight variations in dosimetric metrics to small volumes (i.e., D0.03 cc) appeared as skewed results in percentage differences, and therefore were taken as differences in absolute volume instead. This relationship is due to the sampling of the finite dose grid (2 mm^3^) for smaller volume ROIs.

In addition to inherent uncertainties of Monte Carlo calculations, we found that slight differences in dose reporting also arise between the TPS and TDS during the dose calculation consistency validation, as a result of the lack of partial voxelization and differences in intravoxel interpolation. Currently, MRIdian TPS calculates dose statistics based on the dose grid and then extrapolates to the resolution of the image grid. MRIdian TDS is a finer representation of the dose statistics by sampling the dose solely based on the calculation grid and is therefore independent of the image grid (i.e., no downsampling or extrapolation is applied). While the differences are small, it is possible for dose statistics to report inconsistencies between the TDS and TPS for small volume metrics (i.e., D0.03 cc), which could result in pass/fail criteria to be different for the same fluence.

For the robustness evaluation, while no differences were observed in DVH metrics, there were some differences in the modulation between the TPS and TDS for the same geometry, such as CTV and its derived structure, PTV33 (**Table 4**). Of note, the TDS ART UI utilizes a more efficient leaf sequencer, in which segments are combined when disjointed fluence is present. Therefore, during ART optimization extra modulation often occurs due to the optimizer having a greater number of available unused segments. Since the optimization settings were set to 50 total segments, both TPS and TDS plans generated a resulting plan with roughly 50 segments—hence allowing the TDS ART UI to get more complex. As such, the modified leaf sequencer from the TDS may produce slightly different coverage for larger targets such as the CTV and PTV33. When we observed this during commissioning, we implemented a modulation check during online ART in which the planner reduces the segments until a similar modulation is achieved on the ART plan relative to the TPS baseline plan. For the image value to density validation, we found good agreement between the reported electron density between the TPS baseline plan and the TDS adaptive plan. The purpose of this test was to verify the consistency of electron density values between the two platforms, benchmarking them was not the objective. Of note, we took advantage of a previously acquired CT scan of a dedicated electron density phantom from commissioning for the image value to electron density verification test. Note that a patient scan could have been easily used to verify electron density consistency between the TPS and TDS.

The end-to-end test evaluated the system’s ability to accurately deliver the planned dose in the ART workflow in a single, integrated test. Plan delivery accuracy was evaluated based on a measurement within 2% of the calculated adaptive TDS dose (11). Note that for this test, a large shift was applied to simulate a target interfraction motion beyond the irradiated volume. While an SBRT plan was used, the overall dosimetry was not representative of an ablative technique as intentional homogenous dose distribution was applied across the chamber active volume for statistics. Sharp dose gradients, found in SBRT plans, would lead to difficult measurement conditions in a finite chamber volume, hence the use of a homogenous dose distribution. Moreover, the rationale for the uniform dose distribution was to minimize the effect of sub-millimeter translations, which could result in spurious dose reporting and a failed test. Shifting dose distributions, which are routinely performed on patient QA software to account for sub-mm setup uncertainties, are not available when taking an ionization chamber measurement which are sensitive to a static dose gradient.

We believe that QA procedures should reflect the intervention performed; as such, the tests in this work were designed to be indicative of the UI changes and how the ART program is clinically utilized. Note that the tests and procedures outlined in this work may only need to be applied as a subset based on the specific changes in the UI. Users should work with their vendor to know what aspects of the system were modified in the patch for such evaluations.

One advantage of this work is the methodology of the tests can be implemented as part of an ongoing QA program for online ART. Specifically, the end-to-end tests can be utilized as part of an annual QA program. Furthermore, these tests can be part of the initial commissioning of the system, and baseline tests/plans can be reused during patch installation, which will reduce the estimated FTE hours needed to perform these tests. Other studies have addressed ongoing QA into daily practice; for example, Chen et al. demonstrated daily online QA procedure testing the integration of critical ART components (12).

There are various ways to evaluate the TPS vs. TDS beam model and dose calculation consistency. We performed a 3D gamma analysis between exported DICOM RT dose files of the baseline plan from the TPS and the same fluence calculated on rigid geometry during the ART workflow from the TDS. Not all institutions may have access to the same resources, but there are viable alternative methods available to evaluate beam consistency. Users could superimpose DVH curves on each other, evaluate individual DVH metrics, or run a gamma analysis comparing the dose distribution from the same plan generated by the TPS and TDS.

The scope of this work is related to UI (i.e., software), and not hardware modifications. As such, if hardware components were involved in the system patch and/or upgrade, then further system QA would need to be assessed, including but not limited to imaging and radiotherapy components. Patch and software upgrades influence an ART-capable system’s treatment planning software and the data transfer capabilities between the TPS and TDS. As such, IMRT QA is not a necessary component of a patch upgrade, because the mechanical and beam components of the Linac remain unaltered. QA of radiation production, imaging, and mechanical functionality is recommended if their respective components are altered. Recommendations include performing monthly QA per institutional guidelines (i.e., TG142).

While the ability to accurately deliver adaptive RT is dependent on the gating (13), the QA of intrafraction motion management (14) is outside the scope of this work. Similarly, deformable image registration (DIR) accuracy is a key feature of the online ART workflow. This work assumes baseline performance commissioning of the DIR algorithm (2) has been performed and algorithm remains unaltered in the UI. Additional work related to online ART is the QA of dose accumulation (15), which is also outside the scope of this work.

After a software upgrade is performed, it is recommended that the therapists and qualified medical physicists mode up any active patients as well as confirm any other clinical functionality associated with their institution’s specific ART workflow. If there is no change in the UI or clinical workflow (i.e., software patch for a bug), a re-training is not necessary. Furthermore, if UI or clinical workflow has changed, then it is recommend that therapists be involved with a dry run workflow on phantom. The onus of therapists’ involvement post-QA lies with each ART program, as each institution has different methods of operation and divisions of labor.

There are several limitations to this work. First, this study is a single institution/machine reporting. Additionally, no longitudinal QA assessment was performed. Another limitation of this work is that QA procedures are specific to one ART modality. This study specifically does not outline daily, monthly, and annual QA for ART, although as previously stated, the proposed post-software upgrade ART QA procedures can be adopted and applied as routine QA (i.e., Annual). Future work will be on reporting implementation of routine QA for ART. Specifically, we envision daily QA consisting of a TPS checksum/integrity QA of ART TDS. For monthly QA, we envision a simplified approach to the end-to-end procedures previously described in this work.

Lastly as previously mentioned, QA procedures were developed around institutional workflow protocols (i.e., imaging protocol, planning technique). Another limitation of our study is that QA procedures may need to be adapted to institutional workflow. Specifically, different institutions will have different workflow protocols and will need to design QA procedures to reflect ART program use.

## 5 Conclusion

An online ART QA program for post-software upgrade has been developed and implemented on an MR Linac system. Tests were implemented to validate in online ART workflow: UI configuration, segmentation, beam model, dose calculation algorithm, optimizer robustness, relative electron densities, and end-to-end absolute dose. The practice of online ART continues to grow but remains an emerging paradigm, and currently there is no official standards established by AAPM and/or ASTRO to guide users on quality assurance for adaptive radiotherapy. As such, we have implemented the recommended QA procedures described here after a post-software upgrade. The outlined QA procedures were demonstrated feasible for a low-field MR Linac system. The scope of this work can be applied and adapted broadly to other online ART platforms.

## Data availability statement

The original contributions presented in the study are included in the article/supplementary material. Further inquiries can be directed to the corresponding author.

## Author contributions

NB: Conceptualization, Data curation, Formal analysis, Methodology, Writing – original draft, Writing – review & editing. JB: Conceptualization, Methodology, Writing – review & editing, Supervision. MC: Resources, Writing – review & editing. RK: Writing – review & editing. YW: Writing – review & editing. MM: Supervision, Writing – review & editing. AG: Writing – review & editing, Resources, Supervision. KM: Conceptualization, Formal analysis, Investigation, Supervision, Writing – original draft, Writing – review & editing.
